# Longevity of Aesthetic Fixed Space Maintainers in the Anterior Area of the Pediatric Dental Patient

**DOI:** 10.3390/children10111734

**Published:** 2023-10-26

**Authors:** María Biedma-Perea, Carolina Caleza-Jiménez, Asunción Mendoza-Mendoza, David Ribas-Pérez

**Affiliations:** Department of Stomatology, University of Seville, 41009 Seville, Spain; mbiedma1@us.es (M.B.-P.); amendoza@us.es (A.M.-M.);

**Keywords:** aesthetic maintainers, fixed space maintainer, primary incisor loss, traumatic injuries

## Abstract

Space maintenance for children at very early ages in primary molars or posterior sectors is widely used and the scientific evidence is clear as to its indications. In the anterior sectors there are doubts as to whether there is a loss of space and its use is usually accompanied by aesthetic, phonatory requirements or the completion of certain habits. In this type of aesthetic anterior maintainer, there are many medium and long-term complications that can occur. Purpose: To evaluate the factors determining possible complications capable of reducing the longevity of aesthetic fixed space maintainers placed due to premature loss of temporary upper anterior teeth. Methods: Data were collected on 100 patients of 1–5 years of age requiring fixed space maintainer placement in the upper anterior area due to caries or traumatisms. Results: Complications were recorded in the form of resin tooth fracture (in 41% of the cases), welding fracture (16%), detachments (28%), gingivitis (26%) and root resorptions (8%). Space maintainer placement in younger children (12–24 months of age) was associated with a significantly greater incidence of root resorptions (*p* < 0.05). Conclusions: Within the limits of the present study, it is concluded that fixed space maintainers would be a good treatment option in patients with prematurely missing anterior teeth, though the use of temporary first molars as abutments could imply a greater risk of failure, with a lesser incidence of root resorptions. Periodic checks and adequate measures of hygiene are essential.

## 1. Introduction

The premature loss or extensive damage of the temporary upper anterior teeth poses a challenge for the pediatric dentist due to the young age of the patients and the difficulties in handling their behavior [1]. Such situations are fundamentally a consequence of caries in early childhood or traumatisms, and their aesthetic rehabilitation is considered to be crucial in order to maintain the length and functions of the dental arches [2].

Early childhood caries (ECC) is defined as the presence of one or more decayed (non-cavitated or cavitated lesions), missing or filled (due to caries) surfaces in any primary tooth of a child under 6 years of age. ECC is preventable but currently affects >600 million children worldwide and remains mostly untreated. Early childhood caries often leads to anterior tooth extraction. The prevalence of ECC for children younger than 6 months is reported at 23.8% and children between 36 and 71 months at 57.3% [3].

Premature loss of primary anterior teeth due to trauma can be the outcome of an avulsion, extraction after the injury because of poor prognosis, late complications of the injury or early exfoliation because of accelerated resorption of the root. The prevalence of avulsion out of all types of traumatic injuries to primary teeth ranges between 5.8% and 19.4%. These traumas occur more often in 2–4-year-old children and it affects boys 1.2–1.5 times more often than girls. The maxillary primary central incisor is involved more than any other tooth, followed by maxillary lateral incisors and mandibular central incisors [2].

The presence of the anterior teeth contributes to avoiding the loss of length of the arch in the anterior area, guiding eruption of the subsequent permanent teeth for incisor function. It also helps to preserve the basic functions of the dentition (chewing, swallowing and speech) [4] and avoids the appearance of parafunctional habits [1] and behavioral disorders that have a negative impact upon the social interaction of the children [5,6]. 

A number of authors have proposed solutions for replacing lost primary upper teeth, including the placement of aesthetic fixed space maintainers in the anterior area. The aim of these maintainers is to allow aesthetic restoration and rehabilitation of the abovementioned functions and ensure correct eruption of the permanent incisors [7]. 

**Figure 1:**
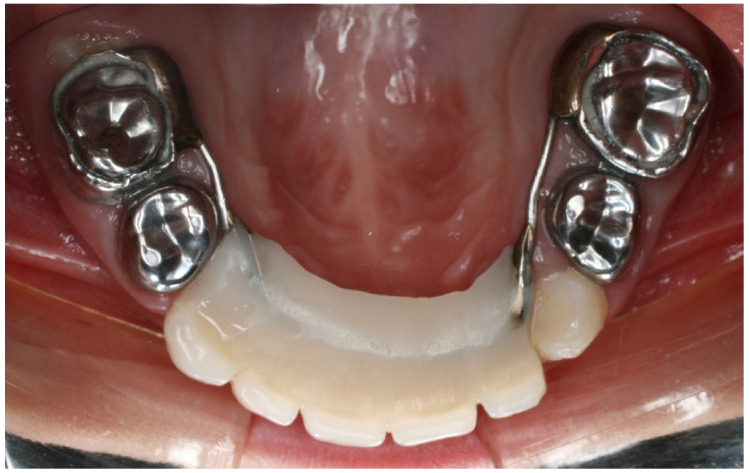
Correct design of a fixed maintainer in the upper anterior teeth of a 3-year-old boy.

In this regard, it is essential for the space to be maintained until eruption of the permanent teeth occurs, without interfering with their normal eruption or with the development of the supporting bone [7,8]. The design should be simple in order to allow correct hygiene, with adequate band or crown adaptation [9] (Figure 1). 

**Figure 2:**
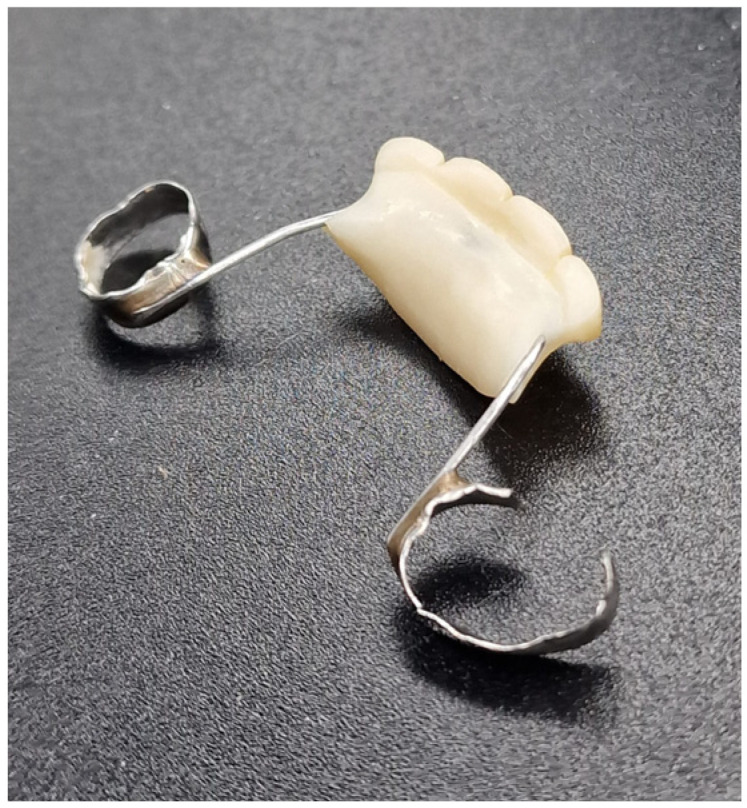
Possible complications of space maintainers: a fracture of the aesthetic fixed space maintainers. Number 3 is root resorption of the anchoring tooth abutment.

**Figure 3:**
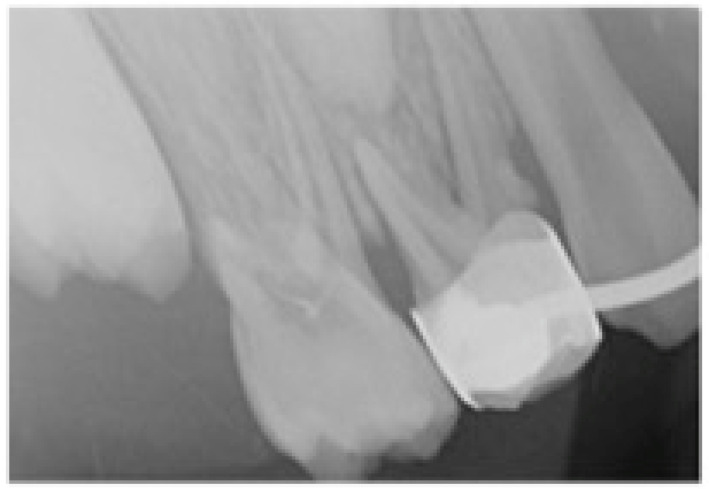
Possible complications of space maintainers: a root resorption of the anchoring tooth abutment.

Athough they are described as an effective and inexpensive treatement [10], a few studies have detailed possible complications: the development of caries, mucosal hyperplasia secondary to friction with the appliance, impingement of the latter, fractures (teeth, welding), decementing of bands and damage to the anchoring teeth such as root resorption or other root disorders [7,9,10] (Figure 2 and Figure 3). 

In view of the few publications available on the subject, the present study was carried out to evaluate the factors determining possible complications capable of reducing the longevity of aesthetic fixed space maintainers in the anterior area. 

## 2. Materials and Methods

### 2.1. Data Collection

Data were collected on pediatric patients seen in a private dental clinic (Coinsol Clinic S.L.) in Seville (Spain) between January 2017 and December 2022. The patients were 63 boys and 37 girls (*n* = 100) of 1–5 years of age and had required the placement of a metal and resin fixed space maintainer in the upper anterior area.

The patients that met all the inclusion criteria and none of the exclusion criteria were assigned a research subject number, and the required study data were obtained from the case histories (Table 1). 

The data were collected using a standardized form that included patient age at the time of placement of the appliance, gender, the cause of tooth loss, the anchoring tooth and its possible pulp treatment, the number of replaced teeth and the appearance and timing of complications (gingivitis, fractures, detachments, resorptions). Each patient was assigned a number and tabulated in an Excel table. We provided the parents/guardians information about this research study. They gave permission for their child to take part and we carried out the study in accordance with the Declaration of Helsinki and with the Ethics Committee of the University of Seville.

### 2.2. Treatment Protocol

All patients were treated by the same operator, and all the space maintainers were manufactured by the same technician following alginate impression (Litochrom^®^, Lascod, Italy) and the production of a positive plaster cast (Vel-mix stone^®^, Kerr, Italy). The design consisted of two metal crowns or two bands over the temporary molars, a 0.9 mm stainless steel wire welded to the bands/crowns and crossing the lingual surfaces of the rest of the teeth and resin teeth bonded to the arc with acrylic resin. After fitting was checked, definitive cementing with glass ionomer (Ketac Molar, 3M ESPE, St. Paul, MN, USA) was carried out. Clinical and radiological checks were performed every 6 months, with a duration of follow-up of 36–48 months. 

### 2.3. Statistical Analysis

Considering the type of variables involved (mostly dichotomic qualitative variables), a descriptive study was carried out, with calculation of the mean and standard deviation (SD) and percentages. Pearson’s chi-square test was used for hypothesis testing, considering a value of *p* < 0.05 as statistically significant. Survival analysis was used to assess the timing of complications (fractures, detachments, etc.), relating the corresponding dichotomic variable (presence or absence of a given complication) to time in order to determine the probability of occurrence of the complication. Since the distribution of the complications was highly asymmetrical, Kaplan–Meier tables and medians were used as the most appropriate descriptive measure. The SPSS version 23.0 statistical package was used throughout. Statistical significance was considered for *p* < 0.05. 

## 3. Results

### 3.1. Sample Characteristics

During the study period (2017–2022), a total of 100 patients of 1–5 years of age received an upper aesthetic space maintainer following premature dental loss due to caries (38%) or traumatism (62%) (Table 2). The mean age of the sample was 2.5 years with an SD of 10.7 months. 

The space maintainer was fitted for the replacement of a single missing tooth in 47 cases, two missing teeth in 25 cases, three missing teeth in 1 case, four missing teeth in 26 cases and six missing teeth in a single patient. The anchoring teeth were mainly first molars (78%), and 22% of the anchoring teeth had received pulp treatment (pulpotomy/pulpectomy) (Table 2). 

During the study, complications were recorded in the form of resin tooth fracture (in 41% of the cases), welding fracture (16%), detachments (28%), gingivitis (26%) and root resorptions (8%) (Table 2). The resin tooth fractures and welding fractures occurred within one year after placement of the space maintainer, between one and two years after placement and in very few cases after this time. 

Over one-half of the detachments and the appearance of gingivitis occurred within one year after placement of the space maintainer. Lastly, of the eight recorded root resorptions, three were identified from the radiographs obtained between one and two years after placement of the space maintainer and five between two and three years after placement (Table 2). 

The complications occurred after different time intervals, ranging from two months when a resin tooth fracture was recorded to sixteen months when root resorption was described (Table 3). 

**Figure 4:**
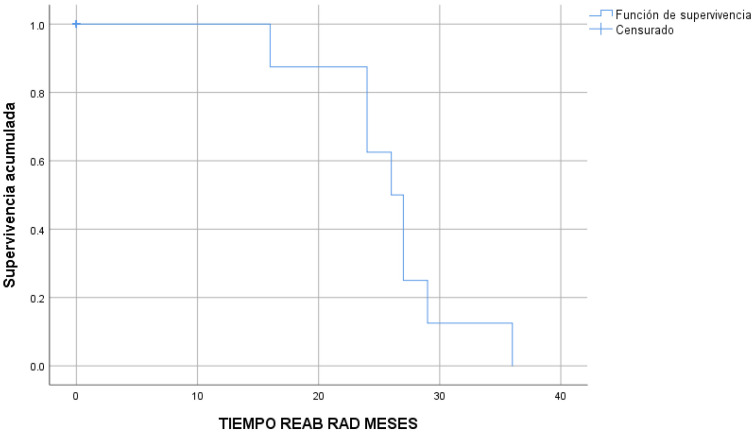
Root resorptions in time (Kaplan–Meier survival estimate).

**Figure 5:**
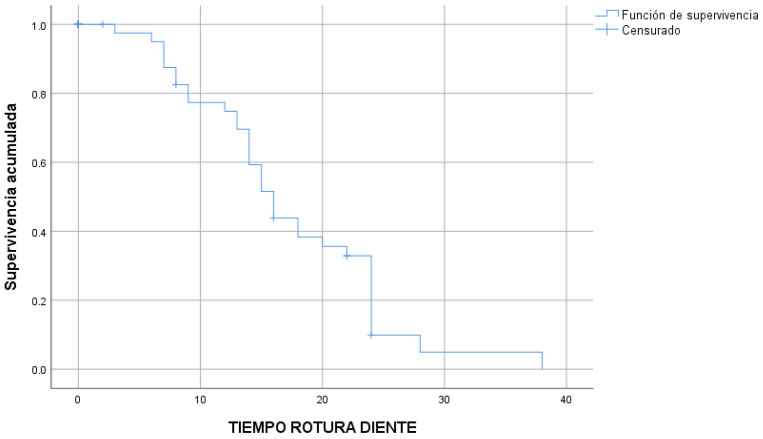
Resin tooth fractures in time (Kaplan–Meier survival estimate).

The eight root resorptions occurred after a median time of 26 ± 1.4 months (Figure 4) and the 35 tooth fractures occurred after a median time of 16 ± 1.0 months (Figure 5). 

**Figure 6:**
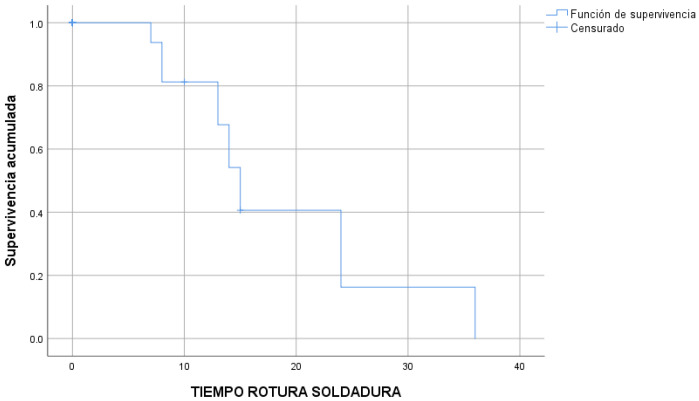
Welding fractures over time (Kaplan–Meier survival estimate).

**Figure 7:**
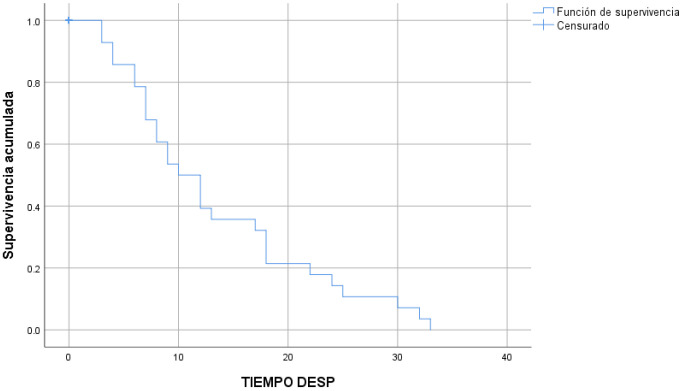
Detachments over time (Kaplan–Meier survival estimate).

The 14 welding fractures occurred after a median time of 15 ± 0.9 months (Figure 6) and the 28 detachments in turn occurred after a median time of 10 ± 1.8 months (Figure 7). 

**Figure 8:**
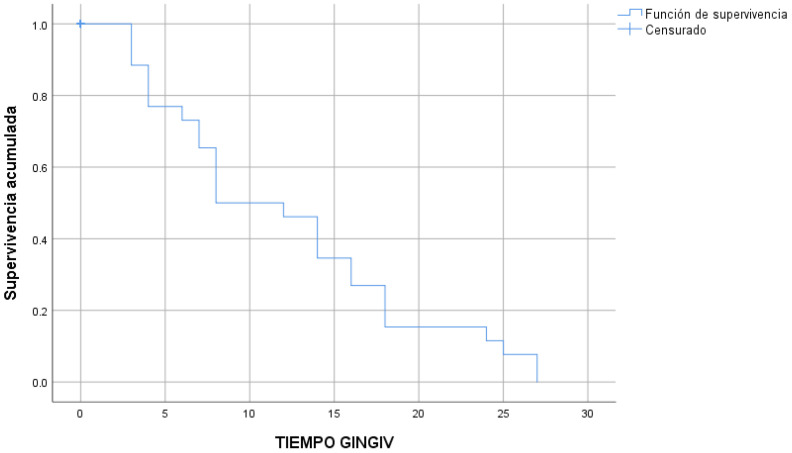
Gingivitis that occurred in the time studied (Kaplan–Meier survival estimate).

Lastly, the 26 cases of gingivitis occurred after a median time of 8 ± 2.3 months (Figure 8). 

### 3.2. Correlation of Variables

**Figure 9:**
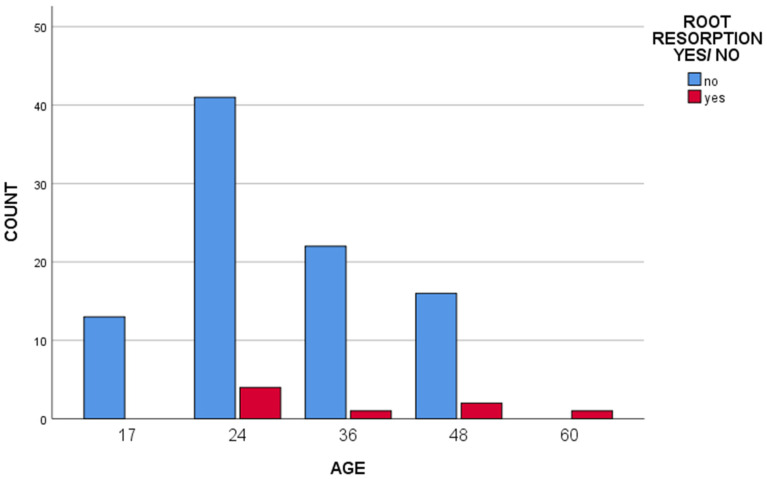
Bar histogram of the correlation between patient age at time of tooth loss and the appearance of root resorptions.

On analyzing the clinical and radiographic findings, the placement of space maintainers in the youngest children (12–24 months of age) was seen to be associated with a significantly greater number of root resorptions (*p* < 0.05) (Figure 9). However, no statistically significant differences in relation to patient age at the time of placement were recorded for any of the other complications. 

**Figure 10:**
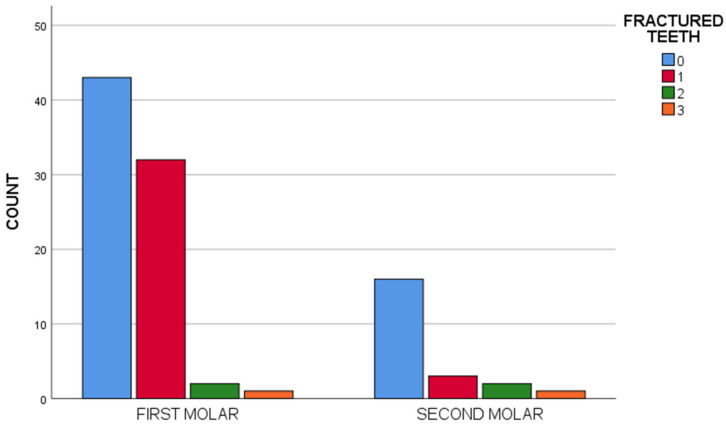
Bar histogram of the correlation between the type of anchoring abutment and resin tooth fracture.

The space maintainer anchoring teeth (first or second molars) showed no statistically significant association (*p* > 0.05) with any of the studied complications, though maintainers anchored to temporary first molars resulted in a greater number of resin tooth fractures (Figure 10).

The application or not of pulp treatments in the anchoring teeth had no significant influence upon the appearance of complications. Likewise, the number of teeth replaced was not significantly related to the appearance of reabsorption, fracture, detachment or gingivitis. 

Table 4 reports the associations between the different variables of the aesthetic fixed space maintainers and the appearance of complications. 

## 4. Discussion

After carrying out this study, we have observed that the appearance of complications very often occurs when aesthetic fixed space maintainers are used. Most cases were replacements of a single tooth due to trauma. The main complications were root resorption, resin tooth fracture, welding fracture, detachments and gingivitis and the youngest children (12–24 months of age) were associated with a significantly greater number of root resorptions (*p* < 0.05).

In many cases, due to early-onset caries or dental traumatisms [11], children may suffer the premature loss of one or more anterior teeth; this in turn can alter the eruption and development of the subsequent permanent tooth or teeth, resulting in malocclusions. Furthermore, self-esteem and socialization of the child may be affected in this very important stage in life [8,12]. The use of aesthetic space maintainers in the anterior area thus must be considered, avoiding malocclusions and parafunctional habits. These appliances must be hygienic, durable and inexpensive [13]. Furthermore, fixed space maintainers require less collaboration on the part of the patient, are better accepted and cause less irritation of the oral tissues than removable appliances [14]. Volpato et al. [10] declared that aesthetic space maintainers were a favorable treatment option regardless of the age of the patient or the number of missing teeth. Removable space maintainers may result in failure if patients fail to use them, are more susceptible to being lost and can affect the soft tissues [11]. 

The use of fixed space maintainers in children could be limited by the modifications of the dental arch, though there is a stable period from 3–5.5 years of age in which the arch and dimensions become consolidated [12]. In the present study, we included smaller children, from 12 months of age, in which premature incisor loss fundamentally resulted from accidental falls when taking their first steps; in some cases, this required the use of the temporary first molars as abutment teeth. We found the youngest children (12–24 months of age) to present a significantly greater number of root reabsorptions (*p* < 0.05). The use of dental prostheses is recommended before three years of age. Early prosthodontics contribute to restoring and normalizing the function of the masticatory muscles and the skeletal growth pattern, reducing the loss of vertical dimension and the tendency towards class III malocclusion [15]. Therefore, we must warn parents of the greater complications in smaller children but the importance of rehabilitation with aesthetic space maintainers.

Periodic long-term follow-up is a limitation of fixed space maintainers and requires patient and parent education and motivation [8]. In the present study, we only evaluated those space maintainers in children that reported every 6 months for the check-up visits over a follow-up period of at least 36 months. Few published studies report such a long period [16,17]. This education is very necessary to diagnose all the possible complications as soon as possible.

Another important aspect for ensuring a higher success rate is careful oral hygiene. The gums are more vulnerable to traumatism during band or crown placement. Furthermore, good oral hygiene is more complicated on the lingual surfaces, due to difficulties of access. Hosseinipour et al. in 2019 [18] recorded a statistically significant association in terms of pocket depth in the mesiolingual and distolingual zone of the abutment teeth. In our study, no correlation was observed between gingivitis and any of the studied variables. Brushing with fluoridated toothpaste and the application of oral rinses 2–3 times a day are advised, supervised by the parents, together with the use of microbrushes or superfloss around the resin teeth, and topical fluor application in the dental clinic [19]. The consumption of probiotics could be used to prevent dental caries and gingival diseases in these cases [20,21].

Adequate retention of the bands or crowns is important for the success of fixed space maintainers and for avoiding detachments. None of our study variables were significantly associated with detachment [21]. Kaur et al. in 2021 [22] reported that retention is conditioned to close adaptation to the tooth by the cement. In 2021, they carried out an in vitro study that evidenced greater detachment resistance with self-adhering resin cements and resin-modified glass ionomer cements than with the usual glass ionomer cements (such as that used in all the cases in this study). Glass ionomer cements have been very popular for cementing fixed space maintainers [23]. They are characterized by low solubility in saliva and great resistance to traction and compression and also form ionic bonds with stainless steel [24]. The disadvantage of this kind of cement is its sensitivity to moisture during setting and the fact that maximum bonding strength is reached after 24 h [25]. Advances have been made in the use of resin-modified glass ionomers and self-adhering resin cements, though more long-term studies are needed [10]. Therefore, it is essential to carry out an adequate technique for the adaptation and cementations of the bands and the use of the most appropriate cements.

Another problem that can arise when placing aesthetic fixed space maintainers is welding fracture or fracture of the resin teeth due to excessive flexibility and lack of support [10]. Our review of the literature identified no studies on these fractures and their correlations to the different anterior fixed space maintainer options. Although we recorded no statistically significant relationship, we did record a greater number of fractured resin teeth when the space maintainers were cemented to the temporary first molars. It is perhaps best to use the second primary molar as abutment whenever possible.

Mention must be made of the limitations we found in seeking to compare our results, since the great majority of studies found in the literature are limited to space maintainers for prematurely lost primary molars [2,16,26]. Few data are available on space maintenance after the premature loss of temporary anterior teeth, and the existing publications are mainly descriptive studies or case reports, characterized by very small samples and short follow-up periods [4,7,8,11,18,27,28]. Thus, there is scant scientific evidence in the literature on the sequelae of the premature loss of temporary anterior teeth and on whether loss of space truly occurs in such scenarios. Based on different studies [29,30], in determining the need for space maintenance in prematurely lost primary anterior teeth, it could be postulated that the loss of space is greater in the upper maxilla than in the mandible, in cases of dental crowding, in very small children and in patients with multiple missing teeth. In our study, however, we included patients in which the placement of an aesthetic fixed space maintainer was decided in response to a single missing tooth, in patients with interincisal diastemas and in children up to 5 years of age. Due to a lack of literature to discuss and compare with this study, we must be cautious with our findings.

Some authors have focused on other complications such as speech difficulties. Kalia et al. in 2018 [4] suggested that rehabilitation of the anterior area in children improves speech function, with important and statistically significant changes in different sounds. Other studies have also evaluated the development or prolongation of non-feeding oral habits such as the use of a dummy or thumb sucking. There is little evidence that an edentulous space corresponding to prematurely lost primary incisors will have long-term effects upon non-feeding oral habits [2]. The premature loss of one or more primary incisors has been described as a possible causal factor for tongue thrusting, but here again there is little supporting evidence. The dental literature does not place much importance on the primary incisors in the chewing of food for digestion. Christensen and Fields in 2013 [28] considered that feeding is not a problem even if the four upper incisors are lost and the child continues to grow normally when receiving an adequate diet. 

It could be very interesting to produce computer-aided design and computer-aided manufacturing (CAD-CAM) aesthetic space maintainers by using intraoral scans, a dental CAD software program and a milling machine [31]. The use of CAD-CAM technology in pediatric dentistry has shown tremendous success in recent years. Improved patient compliance and acceptance of treatments are two main advantages. CAD-CAM technology that is quick, precise and does not require a lot of time may be the best option for pediatric patients [32]. 

Space maintainers that use CAD-CAM or 3D print technology with modern and biocompatible materials are called “digital space maintainers”. The drawbacks of traditional manufacturing could be overcome by using this technology. PEEK polymer materials made from polyetheretherketone have a unique mix of strong mechanical properties and are rigid, opaque and biocompatible [32]. Chemical resistance, high-temperature stability, dimensional stability and a wide range of processing possibilities are all provided by the material. There are some studies of posterior fixed maintainers [33,34,35,36] that showed satisfaction in the young patients. However, there has been no study about aesthetic space maintainers with this procedure. Using it could allow innovative advancements because of its accuracy, aesthetics and possibilities of customization. CAD-CAM space maintainers can be provided for young patients and those with craniofacial disorders, with satisfactory patient compliance and with minimal chairside adjustment [31]. CAD-CAM can be used to create customized space maintainers, especially important for children with craniofacial disorders, as their anatomy may be different from that of typically developing children [37].

Furthermore, more studies based on solid methodological designs are needed to adequately recommend aesthetic fixed space maintainers, involving longer follow-up periods, in order to assess the longevity rates and the appearance of complications. 

## 5. Conclusions

- Fixed space maintainers are a good management option in the case of premature loss of anterior teeth because they offer good aesthetics, are well tolerated and durable, avoid the appearance of malocclusions and contribute to functional rehabilitation.
- An early patient age when placing the space maintainer may pose an increased risk of failure, with a greater incidence of root reabsorptions, so they must be checked every 6 months to ensure the early identification of possible complications.
- Further studies involving larger sample sizes and longer periods of follow-up are needed in order to draw more solid conclusions.

## Figures and Tables

**Table 1:** Inclusion and exclusion criteria.

Inclusion Criteria	Exclusion Criteria
Patient with aesthetic fixed space maintainer due to caries or traumatism	Patients with systemic diseases
Clinical check every 6 months	Follow-up of less than 3 years
Radiological check every 6 months	

**Table 2:** Characteristics of the study sample.

Study Variables	Sample (*n* = 100)	Study Variables	Sample (*n* = 100)
Gende		Detachments	
Male	63%	Yes	28%
Female	37%	No	72%
Age		Gingivitis	
12–24 months	58%	Yes	26%
25–36 months	23%	No	74%
37–48 months	17%		
49–60 months	2%		
Cause		Root resorptions	
Caries	38%	Yes	8%
Traumatism	62%	No	92%
No. teeth replaced		Time of tooth fracture	
1	47%	Less than one year	12%
2	25%	1–2 years	27%
3	1%	2–3 years	1%
4	26%	3–4 years	1%
5	0%		
6	1%		
Anchoring teeth		Time of welding fracture	
First molars	78%	Less than one year	4%
Second molars	22%	1–2 years	10%
		2–3 years	2%
		3–4 years	0%
Pulp treatment of anchoring teeth		Time of detachment	
Yes	22%	Less than one year	17%
No	78%	1–2 years	7%
		2–3 years	4%
		3–4 years	0%
Fractured teeth		Time of gingivitis	
Yes	41%	Less than one year	14%
No	59%	1–2 years	9%
		2–3 years	3%
		3–4 years	0%
Fractured welding		Time of root resorption	
Yes	16%	Less than one year	0%
No	84%	1–2 years	3%
		2–3 years	5%
		3–4 years	0%

**Table 3:** Complications and time of appearance.

Type of Complication	No. of Cases	Time of First Case (Months)	Median Months
Root resorption	8	16	26 (±1.4)
Tooth fracture	35	2	16 (±1.0)
Welding fracture	14	7	15 (±0.9)
Detachment	28	3	10 (±1.8)
Gingivitis	26	3	8 (±2.3)

**Table 4:** Chi-square test correlating the different study variables with the appearance of complications of the fixed space maintainers of the anterior area.

Study Variables	*p*-Value	Study Variables	*p*-Value
Age at placement		Anchoring tooth	
Root resorption	0.010 *	Root resorption	0.831
Resin tooth fracture	0.701	Resin tooth fracture	0.062
Welding fracture	0.384	Welding fracture	0.228
Detachments	0.189	Detachments	0.533
Gingivitis	0.709	Gingivitis	0.210
Pulp treatment of abutment teeth		No. teeth replaced	
Root resorption	0.831	Root resorption	0.844
Resin tooth fracture	0.200	Resin tooth fracture	0.065
Welding fracture	0.279	Welding fracture	0.319
Detachments	0.931	Detachments	0.677
Gingivitis	0.481	Gingivitis	0.060

* significant association (*p* > 0.05).

## Data Availability

The study did not report any data.
